# Expression and functional analysis of *CsA-IPT5* splice variants during shoot branching in *Camellia sinensis*

**DOI:** 10.3389/fpls.2022.977086

**Published:** 2022-08-22

**Authors:** Liping Zhang, Donghui Wang, Lan Zhang, Jianyu Fu, Peng Yan, Shibei Ge, Zhengzhen Li, Golam Jalal Ahammed, Wenyan Han, Xin Li

**Affiliations:** ^1^Key Laboratory of Tea Quality and Safety Control, Ministry of Agriculture and Rural Affairs, Tea Research Institute, Chinese Academy of Agricultural Sciences, Hangzhou, China; ^2^College of Horticulture and Plant Protection, Henan University of Science and Technology, Luoyang, China

**Keywords:** adenylate isopentenyltransferase, AU-rich elements, ATTTA motif, *Camellia sinensis* L., cytokinins, shoot branching, splice variants

## Abstract

Alternative splicing (AS) is a process by which several functional splice variants are generated from the same precursor mRNA. In our recent study, five *CsA-IPT5* splice variants with various numbers of ATTTA motifs in the untranslated regions (UTRs) were cloned. Meanwhile, their transient expression, as well as the expression and functional analysis in the two shoot branching processes were studied. Here, we examined how these splice variants regulate the other three important shoot branching processes, including the spring tea development, the distal branching of new shoots, and the shoot branching induced by 2,3,5-triiodobenzoic acid (TIBA) spraying, and thus unraveling the key *CsA-IPT5* transcripts which play the most important roles in the shoot branching of tea plants. The results showed that the increased expression of 5′ UTR AS3, 3′ UTR AS1 and 3′ UTR AS2 could contribute to the increased synthesis of *t*Z/iP-type cytokinins (CKs), thus promoting the spring tea development. Meanwhile, in the TIBA-induced shoot branching or in the distal branching of the new shoots, *CsA-IPT5* transcripts regulated the synthesis of CsA-IPT5 protein and CKs through transcriptional regulation of the ratios of its splice variants. Moreover, 3′ UTR AS1 and 3′ UTR AS2 both play key roles in these two processes. In summary, it is revealed that 3′ UTR AS1 and 3′ UTR AS2 of *CsA-IPT5* might act as the predominant splice variants in shoot branching of the tea plant, and they both can serve as gene resources for tea plant breeding.

## Introduction

Branching patterns determine the plant architecture and greatly affect key aspects of plant development (Aguilar-Martínez et al., 2007; Martín-Trillo et al., 2011). The sprouting of axillary buds is regulated by a complicated interplay of phytohormones and the growing environment (Tan et al., 2018). The tea plant (*Camellia sinensis*) is an amazing perennial evergreen leaf-used crop (Zhang et al., 2017a; Zhu et al., 2018; Chen et al., 2021). The shoot branching greatly affects the yield, quality and productivity of the tea plant. However, the tea plant has obvious apical dominance and regulation of its lateral branch development is necessary for tea production (Zhang et al., 2017a). Given the importance of shoot branching for the formation of tea plant economic characters, understanding the regulatory mechanism of shoot branching is of great significance (Tan et al., 2018).

Cytokinins (CKs) play key roles in stimulating the sprouting of axillary buds (Tan et al., 2018) and thus promoting shoot branching (Chen et al., 2016; Dierck et al., 2016; Waldie and Leyser, 2018). For example, exogenous CK treatment could promote the outgrowth of axillary buds in several woody plants (Emery et al., 1998; Cook et al., 2001). Endogenous CK is needed for the activation of bud break in apple (Tan et al., 2018). Although CKs are mainly biosynthesized in the root, locally synthesized CKs also play key roles in the shoot branching (Tanaka et al., 2006; Dun et al., 2012; Ni et al., 2015; Chen et al., 2016). For instance, isopentenyladenine (iP) CKs biosynthesized in the stem node could promote axillary bud outgrowth and lateral branch development in tomato (Faiss et al., 1997). The increased number of lateral branches may be caused by the increased CKs in the axillary buds of *Arabidopsis* (Dun et al., 2012).

Isopentenyltransferases (IPTs) catalyze the first and critical step of CK biosynthesis (Zeng and Zhao, 2016; Ishak et al., 2018). There are two types of IPTs, namely, adenylate IPTs (A-IPTs) and tRNA-IPTs (Miyawaki et al., 2006; Tan et al., 2018). There are many kinds of plant endogenous CKs. A-IPTs are probably in charge of most CK synthesis including *trans*-zeatin (*t*Z) and iP CKs (Miyawaki et al., 2006; Ishak et al., 2018; Tan et al., 2018;). Overexpression of the *A-IPT* gene increases branching in the transgenic lines of *Asakura-sanshoo* (Zeng and Zhao, 2016) and chrysanthemum (Tan et al., 2018), whereas *Arabidopsis ipt* mutants show significantly reduced branching (Tan et al., 2018). The outgrowth of axillary buds could be stimulated by up-regulated *A-IPT* expression in axillary buds and stem nodes (Cook et al., 2001). In the pea plant, decapitation markedly induces the transcription of *PsIPTs* in stem nodes and CK accumulation in the stem nodes and axillary buds (Tanaka et al., 2006; Tan et al., 2018).

AS is a course by which several functional splice variants are generated from the same precursor mRNA (Srivastava et al., 2018; Zuo et al., 2019). Regulation of mRNA stability plays a crucial role in the post-transcriptional control of gene expression through producing several splice variants (Voeltz and Steitz, 1998; Gutiérrez et al., 1999, 2002; Narsai et al., 2007). For example, light regulation of mRNA stability has been reported in some plant nuclear and chloroplast mRNAs (Bhat et al., 2004). AU-rich elements (AREs) are 50–150 nt motifs, which are very rich in adenosine and uridine bases and continually happen in the 3′ UTRs of unstable mRNAs (Matsuda et al., 2001; Feldbrügge et al., 2002; Gutiérrez et al., 2002).

In higher eukaryotes, the AUUUA motif and other AREs are the most usual *cis*-acting elements that mediate rapid mRNA decay (Matsuda et al., 2001; Feldbrügge et al., 2002; Gutiérrez et al., 2002), and play leading roles in gene regulation during development and stress response (Ohme-Takagi et al., 1993; Voeltz and Steitz, 1998; Gutiérrez et al., 1999; von Roretz et al., 2011; de Toeuf et al., 2018). For example, in mammals, a number of early-responsive genes are modulated by the instability of mRNAs which contain AUUUA motifs in the 3′ UTR (Voeltz and Steitz, 1998). In *Drosophila* cells, ARE-mediated decay plays a key role in regulating gene expression in different oxygen concentrations (de Toeuf et al., 2018). On the other hand, in plants, the ATTTA motif was also greatly concentrated in transcripts that have short half-life periods (Ohme-Takagi et al., 1993; Feldbrügge et al., 2002; Narsai et al., 2007). For example, the transcript is relatively short-lived as the occurrence of three AUUUA motifs in the 3′ UTR of *par* gene in tobacco (Ohme-Takagi et al., 1993). Mutations of the AUUUA motifs increased the mRNA stability and enhanced accumulation of the M11-fused GFP mRNA in *Arabidopsis* (Voeltz and Steitz, 1998; He et al., 2012), whereas insertion of AUUUA motifs destabilized the reporter transcripts and thus decrease mRNA levels in transgenic plants (Ohme-Takagi et al., 1993; Gutiérrez et al., 1999; Feldbrügge et al., 2002). The regulation of mRNA stabilization could contribute to the clock-regulated transcription of *AtGUTs* which have AUUUA motifs in *Arabidopsis* (Gutiérrez et al., 2002).

There are several shoot branching processes that are important for tea production. For example, firstly, the growth and development of spring tea determine the yield and quality of the famous tea. Secondly, the new shoots of the tea plant are from distal branching, which are similar to that in the one-year-old apple shoots (Cook et al., 2001). Thus, reducing the branching position is necessary for promoting dwarfing of the tea plant. Thirdly, TIBA is a kind of auxin transport inhibitor that suppresses auxin polar transport in plants. We previously found that TIBA application after pruning could promote the formation of productive lateral branches in tea plants (Zhang et al., 2017a). Furthermore, we have found that *CsA-IPT5* splice variants regulate shoot branching stimulated by 6-benzyladenine (6-BA) spraying or pruning in the tea plant (Zhang et al., 2021). Given the key roles of Cs*A-IPT5* transcripts in regulating tea plant shoot branching, here, we further investigated whether and how *CsA-IPT5* splice variants regulate the above other three important processes, and thus find out which splice variants play key roles in the shoot branching of tea plants. The results can recover the molecular mechanism that regulates shoot branching by *CsA-IPT5* splice variants and provide gene resources for the breeding of tea plants.

## Materials and methods

### Plant materials, growth conditions, and treatments

For the three experiments, including spring tea development, exogenous TIBA treatment, and the distal branching habit of new shoots, mature tea plants were used in this study. Especially, in the experiments of exogenous TIBA spraying and the distal branching habit of new shoots, cultivar (cv.) LongJing 43 (LJ43) and cv. Zhongcha 108 (ZC108) which were deeply pruned in the last 10 days of April were adopted, respectively. Furthermore, in the above two studies, the samples collected from 1 m of tea bushes were regarded as a biological repetition, frozen into liquid nitrogen at once, and stored at-80°C for extraction of RNA and protein, and CK detection. The three experiments were all carried out in the tea plantation of Shengzhou experimental base, Tea Research Institute, Chinese Academy of Agricultural Sciences.

### Axillary bud development of the spring tea

In the course of axillary buds developing into young lateral branches, the axillary buds of cv. LJ43 were photographed every 2 days from March 11th to March 23rd, 2019, according to the bud developmental status. The axillary buds which were collected from 10 m of tea bushes were regarded as a biological repetition, frozen into liquid nitrogen, and stored at −80°C for RNA extraction.

### TIBA treatment

As described in our previous report (Zhang et al., 2017a), 100 mg L^−1^ TIBA working solution was prepared by adding solute in distilled H_2_O. In late May, when 1 to 2 leaves below the apical bud outgrew, the tea bushes were sprayed with TIBA. 0.1% (v/v) Tween 20 was added before spraying. Meanwhile, the control tea plants were sprayed with distilled water which contain the same proportion of Tween 20.

### The distal branching habit of tea plant new shoots

The new shoots of tea plants were divided evenly into three sections, including the upper part, middle part, and underpart, according to the length. The number of lateral branches, the blade numbers per lateral branch and the length of lateral branch on the three portions were investigated, respectively. The lateral branches with a length greater than or equal to 1.0 centimeters were regarded as effective lateral branches. At the same time, the branching phenotype of the new shoot was photographed.

### Total RNA extraction and gene expression analysis

Designing and verification of the gene-specific quantitative real-time PCR (qPCR) primers for *CsA-IPT5* splice variants and total transcripts can be found in our recent study (Zhang et al., 2021). Ten primers were designed based on the 5ˊand 3ˊ UTRs of the *CsA-IPT5* transcripts and listed in Table 1. Briefly, 5AS1F, 5AS2F and 5AS3F are the forward primer designed for detecting 5′ UTR AS1, 5′ UTR AS2 and 5′ UTR AS3, respectively. 5ASR is the reverse primer for detecting different AS in 5′ UTR, and the different forward primers all combine with it, respectively. On the other hand, 3ASF is the forward primer for detecting the expression of different 3′ UTR AS and the total expression of *CsA-IPT5*. 3AS1R, 3AS2R and 3AS4R are the reverse primers for detecting 3′ UTR AS1, 3′ UTR AS2 and the total expression of *A-IPT5*, respectively. The different reverse primers all combine with 3ASF, respectively. *CsGAPDH* was used for the housekeeping gene.

**Table 1:** Primer sequences used for quantitative real-time PCR (qPCR) of CsA-IPT5 splice variants and housekeeping gene of tea plant.

Primer	5'→3'	PCR product (bp)
A-IPT 5AS1F	GTTGCATCCGTGATATTTAAGGT	A-IPT 5AS1F + A-IPT 5ASR = 247
A-IPT 5AS2F	GCATCCGTGATATTTAAGCAGGT	A-IPT 5AS2F + A-IPT 5ASR = 247
A-IPT 5AS3F	GCTTTTTCACAATCTGTCAGCTG	A-IPT 5AS3F + A-IPT 5ASR = 249
A-IPT 5ASR	GCATTTTGTCCGAGTTTATAATCTC	
A-IPT 3ASF	CATCATCGTGGACCGGTT	
A-IPT 3AS1R	CCTCCAACCCATAATCATTATACTT	A-IPT 3ASF + A-IPT 3AS1R = 427
A-IPT 3AS2R	GTCAAAAAACGAACCCACTTCC	A-IPT 3ASF + A-IPT 3AS2R = 304
A-IPT 3AS4R	GGTGTTTTGTGAGGTCTCTATC	A-IPT 3ASF + A-IPT 3AS4R = 199
CsGAPDH F	GAGACTGGAGCCGAATTCATT	
CsGAPDH R1	GATCTGGCTTGTAATCCTTCTCA	CsGAPDH F + CsGAPDH R1 = 166

As described in our recent report (Zhang et al., 2021), total RNA from tea plant tissues was extracted and reverse transcribed. The qPCR assay was performed using the ABI 7500 Real-Time PCR system (Applied Biosystems, Foster City, CA, United States) with Power SYBR Green PCR Master Mix. Transcript abundance was normalized to *CsGAPDH*. The relative gene expression and the expression ratio of each splice variation was computed (Livak and Schmittgen, 2001). Six independent biological replicates were performed for each treatment.

### Western blot analysis

The preparation of CsA-IPT5-specific polyclonal antibody was described in our recent report (Zhang et al., 2021). In brief, the total protein of tea tissues was extracted and quantified. Then, the protein solutions were separated by 10% SDS-PAGE gel and transferred onto PVDF membranes. The primary and secondary antibodies were hybridized successively, and the signal was detected (Zhang et al., 2021).

### Measurement of iP/*t*Z-type CK contents

The measurement of iP/*t*Z-type CK contents was carried out by Shanghai Applied Protein Technology Co. Ltd., China (Zhang et al., 2021). To be specific, the samples were ground in liquid nitrogen. 50 ± 3 mg samples were placed into 2 ml centrifuge tubes, and then 50 μl internal standard solution and 1 ml acetonitrile water solution(1% FA)were added orderly. Shock blending for 2 min. Extraction at 4°C and in the dark for 12 h, and centrifugation at 14,000 × *g* for 10 min. 800 μl supernatant was taken and dried with nitrogen gas. The samples were re-dissolved with 100 μl water-acetonitrile (1:1, v/v), followed by centrifugation at 14,000 × *g* for 10 min. The supernatant was taken and analyzed. The detection was carried out using Agilent 1,290 Infinity LC Ultra Performance Liquid Chromatography (UHPLC, Agilent) and 5,500 QTRAP mass spectrograph (MS, AB SCIEX; UHPLC–MS).

### Statistical analysis

The data were statistically analyzed using SAS 8.1 software package (SAS Institute Inc., Cary, NC, United States). Especially, data were analyzed using one-way ANOVA; if the ANOVA analysis was significant (*p <* 0.05), Duncan’s multiple range test was used to detect significant differences among groups. Differences in the treatment as compared to the control were detected by Student’s *t*-tests.

## Results and discussion

Shoot branching is a major factor in plant architecture that affects crop productivity and quality (Zhang et al., 2017a, 2017b). Artificially regulation of branch development is essential for tea planting as its obvious apical dominance (Zhang et al., 2017b). ATTTA motif, which is a kind of the most usual ARE, in the UTRs can regulate the stabilization, transcription and translation of the mRNA (Vincenti et al., 1994; Hyodo et al., 2006). A-IPTs play crucial roles in the biosynthesis of iP/*t*Z CKs (Miyawaki et al., 2006; Wang et al., 2020). As reported in our recent study, five splice variants of *CsA-IPT5* were cloned using the RACE method, and their transcript levels in the multiple tissues and regulatory effects in two shoot branching processes were studied (Zhang et al., 2021). Here, the expression and functional analysis of *CsA-IPT5* splice variants during the other three shoot branching processes were further carried out.

### Involvement of *CsA-IPT5* transcripts in the spring tea development

**Figure 1:**
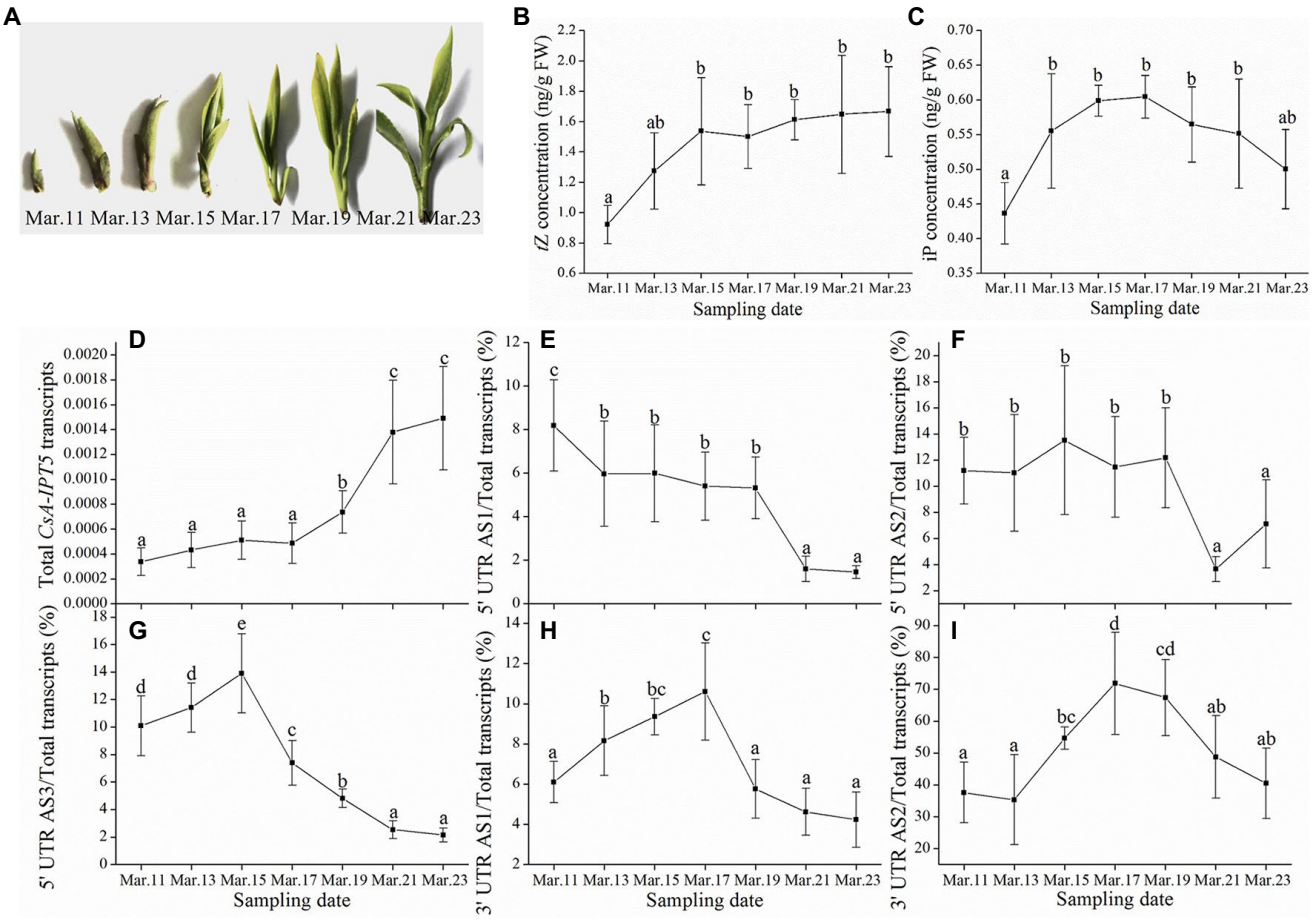
The phenotypes of the axillary buds, cytokinin (CK) concentrations, and the transcriptional levels of *CsA-IPT5* transcripts during the course of spring tea development. The phenotypes of the axillary buds **(A)** and CK concentrations in the axillary buds **(B–C)** during the process of spring tea development. The transcription levels of total *CsA-IPT5* transcripts **(D)** and the expression ratio of each *CsA-IPT5* splice variation **(E–I)** during the process of spring tea development. Data are means ± SD (*n* = 6). For each figure, letters represent marked differences in the index among different time points (*p <* 0.05, Duncan’s multiple range test).

Figure 1A showed the course of spring tea development, every 2 days from March 11th to March 23rd, 2019. With axillary bud development, the concentrations of *t*Z and iP in the axillary bud both showed gradual decreasing trends, respectively (Figures 1B,C). The qPCR analysis suggested that the total *CsA-IPT5* transcripts increased continuously during the process of axillary bud development. The ratio of 5′ UTR AS1 and 5′ UTR AS2 both decreased continuously. Whereas, the ratio of 5′ UTR AS3, 3′ UTR AS1 and 3′ UTR AS2 all showed a trend of first increase and then decrease. More specifically, during spring tea development, the maximum values of the ratio of 5′ UTR AS3, 3′ UTR AS1 and 3′ UTR AS2 reached 13.9, 10.6, and 71.8%, respectively (Figures 1D–I). In short, the above results showed that for the spring tea, the increased transcript of the total *CsA-IPT5* transcripts, as well as the ratio of 5′ UTR AS3, 3′ UTR AS1 and 3′ UTR AS2 could contribute to the increased biosynthesis of *t*Z and iP, thus promoting axillary bud development.

### Involvement of *CsA-IPT5* transcripts in the shoot branching induced by TIBA spraying

We previously reported that the number of axillary buds increased after TIBA spraying, thus stimulating productive lateral branch production in tea plants (Zhang et al., 2017a). Here, the expression patterns of *CsA-IPT5* splice variations were analyzed to further study the regulation mechanism of the TIBA-induced shoot branching process.

**Figure 2:**
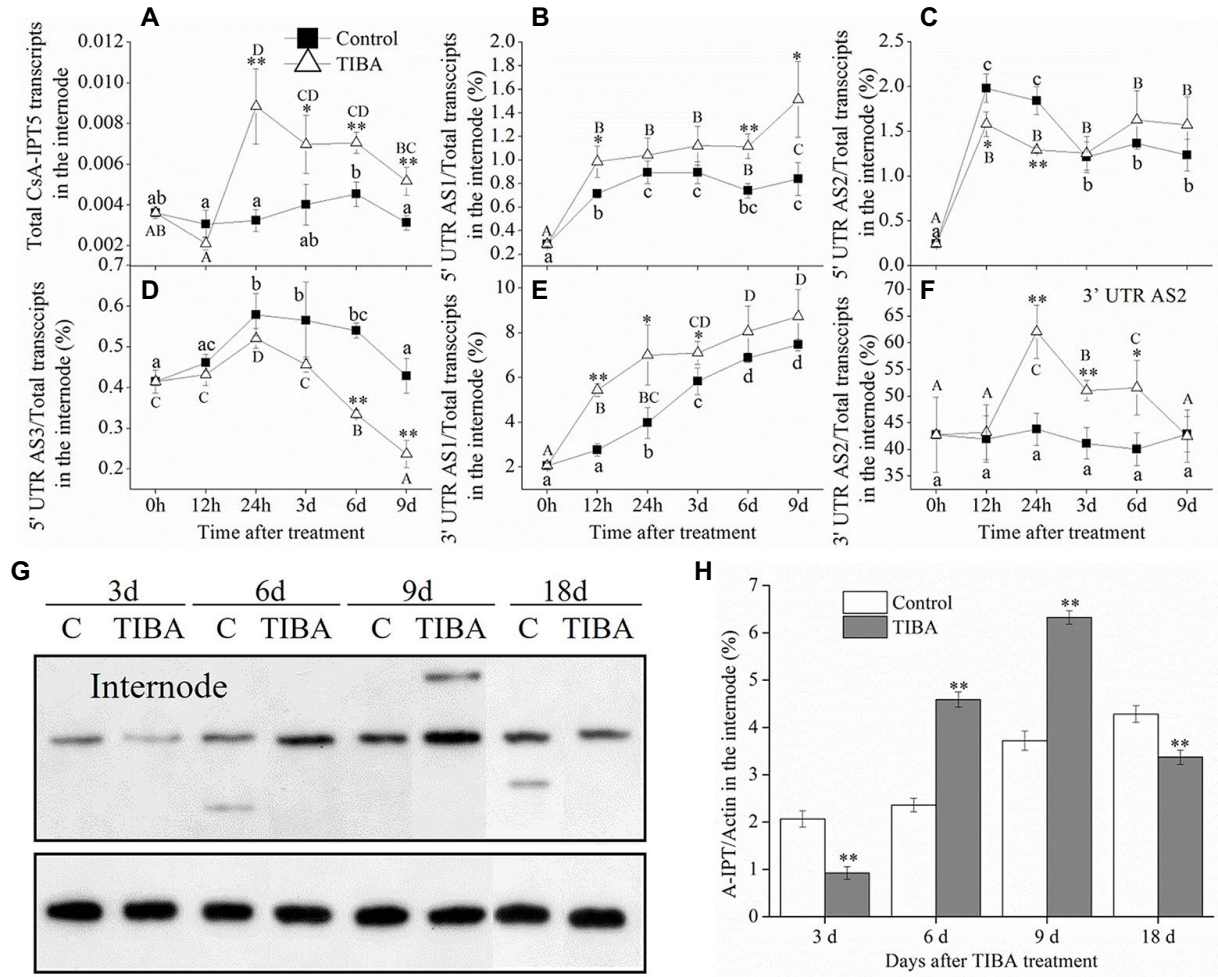
Expression levels of *CsA-IPT5* transcripts and CsA-IPT5 protein in the internode at different time points stimulated by exogenous TIBA spraying. The expression levels of total *CsA-IPT5* transcripts **(A)** and the expression ratio of each *CsA-IPT5* splice variation **(B–F)** in the internode at different time points stimulated by TIBA spraying. Western blot **(G)** and ‘CsA-IPT5/Actin’ **(H)** display the CsA-IPT5 protein expression in the internode induced by TIBA. Data are means ± SD (*n* = 3 or 6). For each figure, asterisks show marked differences in each index between TIBA treatment and the control at each time point (**p < 0.01; Student’s *t*-test).

The result showed that, in the internode, TIBA treatment improved the transcription of total *CsA-IPT5* transcripts, 5′ UTR AS1, 3′ UTR AS1, and 3′ UTR AS2 as compared to the control. Specifically, in the internode, the total *CsA-IPT5* transcripts stimulated by TIBA were all markedly higher at 24 h, 3 days, 6 days, and 9 days relative to the controls. At 12 h, 6 days, and 9 days, compared with the controls, the 5′ UTR AS1 ratio in the internode stimulated by TIBA increased 38.2, 50.6, and 80.4%, respectively. At 12 h, 24 h, and 3 days, compared with the controls, the 3′ UTR AS1 ratio in the internode induced by TIBA increased 97.2, 76.1, and 21.8%, respectively. At 24 h, 3 days, and 6 days, compared with the controls, the 3′ UTR AS2 ratio in the internode induced by TIBA increased 41.8, 24.1, and 28.8%, respectively. On the other hand, TIBA treatment did not significantly increase the 5′ UTR AS2 ratio and 5′ UTR AS3 ratio in the internode relative to the controls (Figures 2A–F).

**Figure 3:**
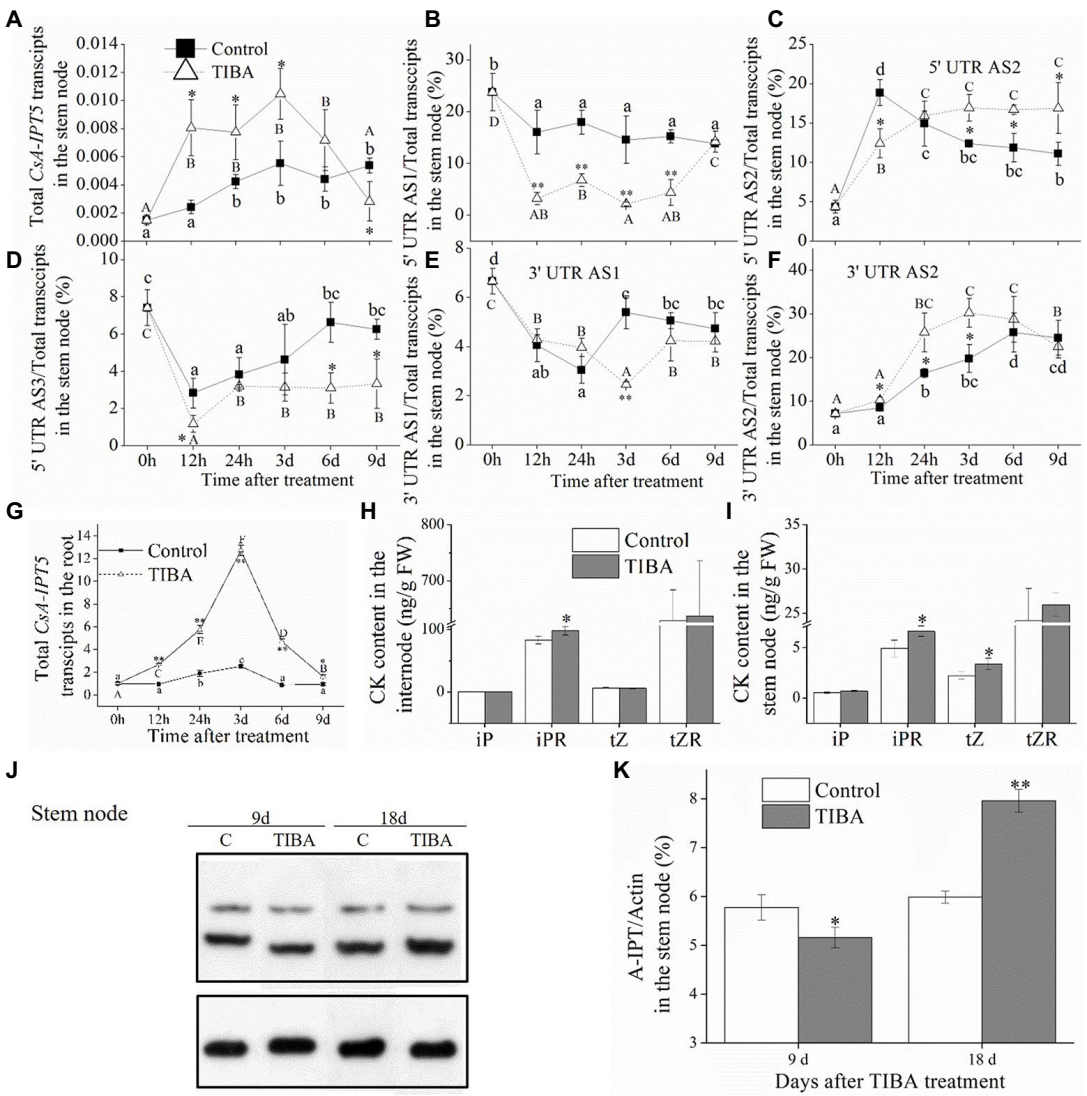
CKs concentrations in the internode and stem node, expression levels of *CsA-IPT5* transcripts and CsA-IPT5 protein in the stem node and root induced by TIBA spraying. Expression levels of total *CsA-IPT5* transcripts in the stem node or root at different time points stimulated by TIBA **(A,G)**. The ratios of *CsA-IPT5* splice variations in the stem node at different time points stimulated by TIBA **(B–F)**. CK concentrations in the internode and stem node stimulated by TIBA **(H,I)**. Western blot **(J)** and ‘CsA-IPT5/Actin’ **(K)** display CsA-IPT5 protein expression in the stem node at 9 and 18 days after TIBA spraying. Data are means ± SD (*n* = 3 or 6). Except for panels **(H,I)**, asterisks show marked differences in each index between control and TIBA spraying at each time point; for panels **(H,I)**, asterisks show marked differences in each index between control and TIBA spraying (**p < 0.01; Student’s *t*-test). In Figures **(A–G)**, letters show significant differences for each treatment among different time points (*p <* 0.05, Duncan’s multiple range test).

In the stem node, the total *CsA-IPT5* transcripts all increased at first and then decreased in control and TIBA spraying, respectively. The transcription of total *CsA-IPT5* induced by TIBA was all markedly higher than that in the controls at 12 h, 24 h and 3 days (Figure 3A). TIBA treatment significantly increased the 5′ UTR AS2 ratio and 3′ UTR AS2 ratio in the stem node relative to the controls. Specifically, at 3, 6, and 9 days, compared with the controls, the 5′ UTR AS2 ratios in the stem node stimulated by TIBA increased 36.7, 40.7, and 52.1%, respectively. At 12 h, 24 h and 3 days, compared with the controls, the 3′ UTR AS2 ratios in the stem node stimulated by TIBA spraying increased 20.4, 56.6, and 52.9%, respectively. On the other hand, TIBA treatment did not increase the 5′ UTR AS1 ratio, 5′ UTR AS3 ratio and 3′ UTR AS1 ratio in the stem node relative to the controls (Figures 3B–F).

In the root, the transcriptional levels of the total *CsA-IPT5* transcripts in TIBA spraying were all markedly higher than that in the control at all time points, respectively (Figure 3G). Compared to the control, TIBA treatment induced the increase of iPR content in the internode and the increase of iPR/*t*Z contents in the stem node (Figures 3H,I). TIBA treatment increased the expression levels of CsA-IPT5 protein in the internode at 6 and 9 days relative to the control (Figures 2G,H). However, at 18 days after TIBA treatment, the expression levels of CsA-IPT5 protein in the stem node were higher relative to the control (Figures 3J,K).

Given that the CK pathway acts as the secondary signaling of the auxin pathway in plant shoot branching (Tanaka et al., 2006; Waldie and Leyser, 2018), this study showed that the auxin transport pathway was dependent on *CsAIPT5* transcripts-dependent CK biosynthesis in the shoot branching of tea plants. There are significant associations among the increased expression levels of *CsA-IPT5* transcripts (including total *CsA-IPT5*, 5′ UTR AS1, 3′ UTR AS1, 3′ UTR AS2), increased CsA-IPT5 protein expression, and improved iPR contents in the internode stimulated by TIBA spraying. Meanwhile, after TIBA treatment, the improved iPR contents in the internode may be also transported from the root, as the elevated transcript levels of total *CsA-IPT5* in the root were detected relative to the control. TIBA also stimulated the expression of total *CsA-IPT5*, 5′ UTR AS2, 3′ UTR AS2, and CsA-IPT5 protein in the stem node relative to that in control, thus increasing the biosynthesis of iPR and *t*Z in the stem node. Therefore, it can be presumed that after TIBA spraying, iPR and *t*Z were locally biosynthesized in the stem node, iPR was transported from the internode and probably from the root were all transported into the axillary bud to stimulate its growth and development (Figure 5B).

Our recent study showed that in the 6-BA-induced shoot branching of tea plants, the transcription of 5′ UTR 5AS1, 5′ UTR AS2, 3′ UTR AS1, 3′ UTR AS2 in the internode all played key roles, whereas 6-BA did not induce the expression of *CsAIPT5* transcripts in the stem node (Zhang et al., 2021). Combined with the above results here, we can see that the transcription of 5′ UTR AS1, 3′ UTR AS1, and 3′ UTR AS2 in the internode all played key roles in the 6-BA and TIBA-induced shoot branching. The transcription of 5′ UTR AS2 and 3′ UTR AS2 in the stem node play key roles in the TIBA-induced shoot branching. Moreover, exogenous application of 6-BA and TIBA both induced the expression of total *CsA-IPT5* in the root compared with the control, whereas the expression patterns of *CsA-IPT5* splice variations in the root were not detected.

### Regulatory effects of *CsA-IPT5* transcripts in the distal branching habit of tea plant new shoots

**Figure 4:**
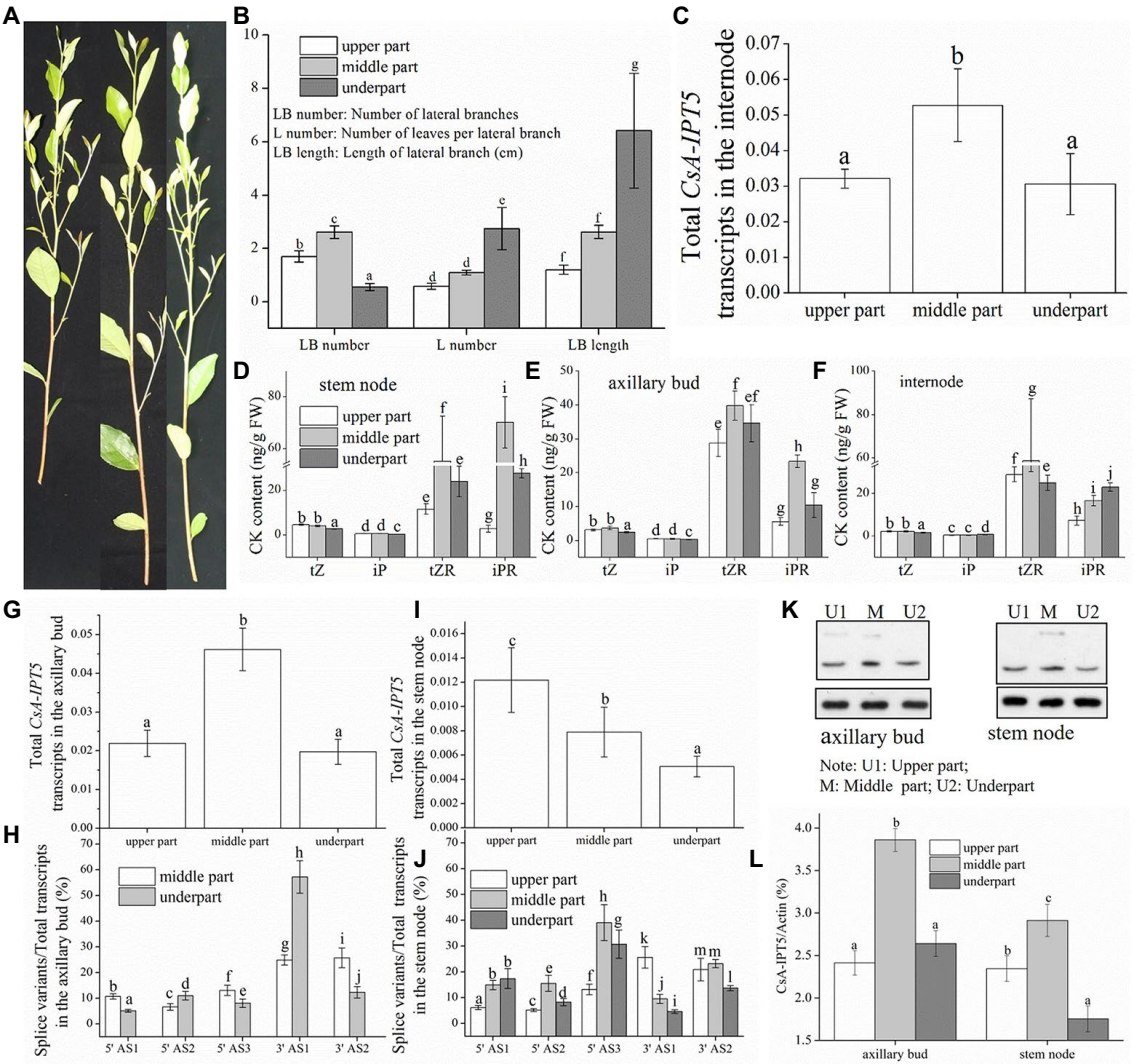
The branch phenotype, CK contents and the expression levels of *CsA-IPT5* transcripts and CsA-IPT5 protein in the different tissues of three positions of the tea plant new shoot. The branch phenotype **(A,B)** and CK contents in the three tissues **(D–F)** of three positions of tea plant new shoot. **(C,G–J)** Expression levels of *CsA-IPT5* transcripts in the three tissues of three positions of the new shoot. Western blot **(K)** and ‘CsA-IPT5/Actin’ **(L)** display the expression levels of CsA-IPT5 protein in the axillary bud and stem node in the three positions of the new shoot. Data are means ± SD (*n* = 3 or 6). For each figure, letters show marked differences in each index or each tissue among three positions of the new shoot (*p <* 0.05, Duncan’s multiple range test).

Figure 4A shows the branching phenotype of the tea plant new shoot. The results showed that the leaves number per lateral branch (L number) and the lateral branch length (LB length) in the underpart were both significantly greater compared with the other two parts, respectively, and there was no marked difference between that in the other two parts (Figure 4B). The fact that the L number and the LB length could represent the outgrowth time of axillary bud in the same plant. Thus, it can be concluded that the axillary buds locate in the lower shoot break firstly relative to that in the other two parts. Furthermore, it is important that the lateral branches number (LB number) in the middle shoot is the highest, whereas it is the smallest in the lower shoot (Figure 4B). Thus, the new shoots of the tea plant are from distal branching.

In lupin, compared with the upper and basal parts, there are fewer lateral branches in the middle of the main stem, and it is attributed to axillary branches (upper, middle and basal of the plant) which elongate at much different rates (Emery et al., 1998). Cook et al. (2001) reported that one-year-old apple shoots are distal branching and there was a greater increase in CK content of distal xylem sap and wood. Here, we study if the distal branching of the tea plant is dependent on *CsA-IPT5* transcripts-dependent CK biosynthesis. In famous tea production, tea plants are normally pruned twice yearly. The first pruning is carried out after spring tea picking, and the second pruning is made around 20 July (Zhang et al., 2021). Here, in the middle of July 2019 before the second pruning, the branching phenotype of the tea plant’s new shoots was analyzed.

The new shoots were equally divided into three portions according to the length. The results showed that there were relatively higher *t*ZR contents in the middle stem node relative to that in the underpart, and there were no significant differences between that in the upper part and underpart. The iP and *t*Z contents in the lower stem node both were lower than that in the other two parts. The iPR content in the upper, lower, and middle stem nodes increased successively (Figure 4D); the contents of *t*Z and iP in the upper and middle axillary buds both were higher than those in the underpart. The iPR content in the middle axillary bud was relatively higher than that in the underpart (Figure 4E); the *t*Z content in the upper and middle internode was both higher than that in the lower internode. The *t*ZR content in the lower, upper, and middle internode increased successively (Figure 4F).

The qPCR analysis suggested that the total *CsA-IPT5* transcripts in the middle axillary bud were higher than that in the underpart. The ratio of 5′ UTR AS1, 5′ UTR AS3, and 3′ UTR AS2 in the middle axillary bud were all relatively higher than that in the underpart, respectively (Figures 4G,H). On the other hand, the total *CsA-IPT5* transcripts and 3′ UTR AS1 ratio in the stem node of the underpart, middle, and upper part increased successively. The 5′ UTR AS2 ratio and 5′ UTR AS3 ratio in the upper, under, and middle stem nodes increased successively. The 3’ UTR AS2 ratio in the underpart stem node was both markedly lower than that in the other two portions (Figures 4I,J). Lastly, the expression levels of the total *CsA-IPT5* transcripts in the middle internode were relatively higher than that in the underpart internode (Figure 4C).

The expression levels of CsA-IPT5 protein in the middle axillary bud were relatively higher than that in the underpart, and there were no significant differences between that in the upper and underpart axillary bud. The CsA-IPT5 protein expression increased successively in the under, upper, and middle stem nodes (Figures 4K,L).

**Figure 5:**
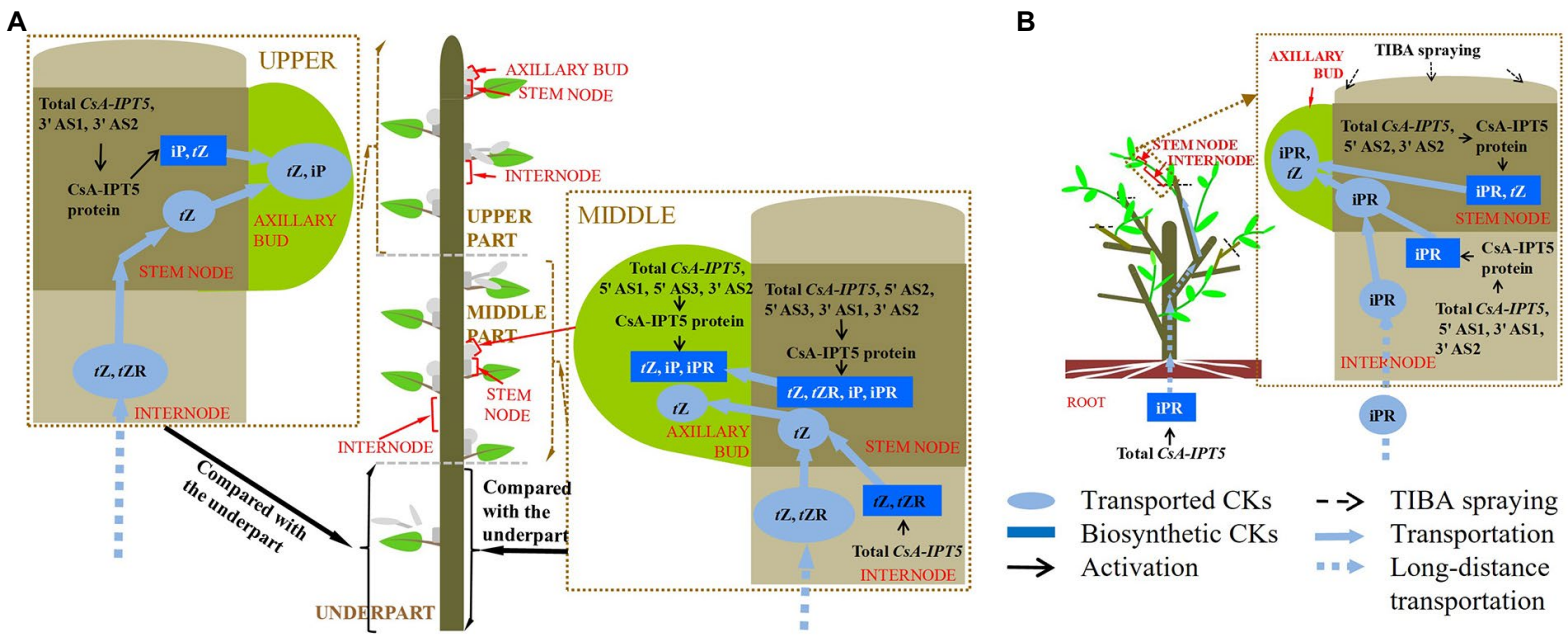
A proposed model of *CsA-IPT5* transcripts in the regulation of the distal branching habit of new shoots **(A)** and shoot branching stimulated by TIBA spraying **(B)** in tea plants. Each shoot branching process regulated by *CsA-IPT5* transcripts is included in a single rectangle surrounded by brown dashed lines, respectively. Light blue ellipses represent the transported CKs. Blue rectangles represent locally biosynthesized CKs. Black arrows represent activation. Black dashed arrows represent exogenous TIBA spraying. Light blue solid arrows represent transportation, whereas light blue dashed arrows represent long-distance transportation.

In short, the differences between the underpart and the middle part were analyzed firstly. The above results showed that compared with the underpart, there are significant associations among the relatively high expression of total *CsA-IPT5* transcripts, 5′ UTR AS2, 5′ UTR AS3, 3′ UTR AS1 and 3′ UTR AS2, relatively high contents of *t*Z, *t*ZR, iP, and iPR, and relatively high CsA-IPT5 protein expression in the middle stem node. Therefore, relative to the underpart, the high contents of *t*Z, *t*ZR, iP and iPR in the middle stem node at least were partly self-synthesized. Second, compared with the underpart, there are significant associations between the relatively high contents of *t*Z and *t*ZR and the increased expression levels of total *CsA-IPT5* in the middle internode. Therefore, the high contents of *t*Z and *t*ZR in the middle internode may be at least partly biosynthesized by itself. Third, compared to the underpart, as the higher expression levels of total *CsA-IPT5* transcripts, 5′ UTR AS1, 5′ UTR AS3, 3′ UTR AS2 and CsA-IPT5 protein in the middle axillary bud, it can be concluded that the relatively high contents of *t*Z, iP and iPR in the middle axillary bud at least was partly biosynthesized by itself. Meanwhile, they may also be partly transported from other tissues, including the stem node, internode and root, thus promoting the faster growth and development of the middle axillary bud (Figure 5A).

On the other hand, the differences between the underpart and the upper part were also analyzed. Compared with the underpart, there are significant associations among the relatively high expression levels of total *CsA-IPT5* transcripts, 3′ UTR AS1, 3′ UTR AS2 and CsA-IPT5 protein, and relatively high contents of *t*Z and iP in the upper stem node. Thus, the higher contents of *t*Z and iP in the upper stem node at least were partly locally-synthesized. Second, it could be concluded that the raised contents of *t*Z and *t*ZR in the upper internode were contributed by potential transport from the root, as there were no marked differences in the transcript levels of total *CsA-IPT5* between that in the upper and that in the under internode. Third, there were no marked differences in the expression levels of total *CsA-IPT5* transcripts and CsA-IPT5 protein between that in the upper and under axillary bud. Therefore, it can be speculated that compared with that at the under shoot, the higher lateral branch number at the upper shoot might be closely related to the relatively high concentrations of *t*Z and iP in the axillary bud, which must be transported from other tissues, including the upper stem node and the root (Figure 5A). Together, these results can provide a fundamental basis for reducing the branching position and thus promoting dwarfing of tea plants.

In summary, based on our recent study (Zhang et al., 2021), the current study further investigated the *CsA-IPT5* AS-regulated post-transcriptional mechanisms of shoot branching. The results showed that in the above three shoot branching processes, the five splice variants may play regulatory roles through complementation or competition with each other. We can see that there are significant associations among the specific *CsA-IPT5* transcripts, including the total transcript, 3′ UTR AS1 and 3′ UTR AS2, contents of *t*Z and iP CKs, and CsA-IPT5 protein expression in the three shoot branching processes, including spring tea development, TIBA-induced shoot branching, and distal branching of new shoots in tea plant.

There are reports in animals that were similar to this study, for example, de Toeuf et al. (2018) reported that metabolic adaptation to oxygen changes depends on the modulation of gene transcription, enzyme activity, and ARE-mediated mRNA stability in *Drosophila* cells. Hyodo et al. (2006) reported that in human mesangial cells, the long form of *high-glucose-regulated* (*HGRG-14*) transcript, which has seven ATTTA motifs, is generated and closely related to decreased HGRG-14 protein levels at high-glucose contents, while a truncated, more stable mRNA is generated under low-glucose condition.

Furthermore, in the shoot branching, except for total transcripts, the *CsA-IPT5* gene was demonstrated to play important roles through transcriptionally regulating ratios of its five splice variations in the several shoot branching processes of the tea plant. The key determinants may be the specific functions that 3′ UTR AS1 and 3′ UTR AS2, which are the predominant transcripts, confer as well as the dynamic equilibrium of gene expression among the five AS transcripts. This study emphasizes the AS-based post-transcriptional regulatory mechanisms in tea plant shoot branching. Together with our recent study (Zhang et al., 2021), this study indicated that the five *CsA-IPT5* splice variants showed differential spatio temporal expression patterns, and the 3′ UTR AS2 could act as the predominant transcript which regulates the shoot branching of the tea plant.

## Conclusion

The 5′ UTR AS3, 3′ UTR AS1 and 3′ UTR AS2 played key roles in the axillary bud development of spring tea. Meanwhile, the 3′ UTR AS1 and 3′ UTR AS2 both regulated the synthesis of CsA-IPT5 protein and *t*Z/iP-type CKs in the shoot branching induced by TIBA application or in the distal branching of tea plant new shoots. In summary, the results suggest that the 3′ UTR AS1 and 3′ UTR AS2 are the predominant transcripts and both play key roles in the three shoot branching processes tested in this study. Combined with our recent study (Zhang et al., 2021), this study proved again that the 3′ UTR AS2 could act as the predominant transcript. However, the interaction between auxin signaling and *CsA-IPT5* transcripts-dependent CK biosynthesis should be deeply studied during the shoot branching of tea plants. Moreover, in the TIBA-and 6-BA-induced shoot branching of the tea plant, the roles of *CsA-IPT5* transcripts-dependent CK biosynthesis in roots should also be studied in greater detail.

## Data availability statement

The raw data supporting the conclusions of this article will be made available by the authors, without undue reservation.

## Author contributions

LiZ, XL, and WH conceived and designed the research, and wrote and revised the manuscript. DW, LiZ, LaZ, JF, PY, SG, and ZL performed the experiments and analyzed the data. GA revised the manuscript. LiZ and DW contributed equally. All authors contributed to the article and approved the submitted version.

## Funding

This work was supported by Zhejiang Province Public Welfare Technology Application Research Project (LGN21C020005), the National Key R&D Program of China (2020YFD1000700), and the Innovation Project of the Chinese Academy of Agricultural Sciences (CAAS-ASTIP-2015-TRICAAS).

## Conflict of interest

The authors declare that the research was conducted in the absence of any commercial or financial relationships that could be construed as a potential conflict of interest.

## Publisher’s note

All claims expressed in this article are solely those of the authors and do not necessarily represent those of their affiliated organizations, or those of the publisher, the editors and the reviewers. Any product that may be evaluated in this article, or claim that may be made by its manufacturer, is not guaranteed or endorsed by the publisher.
